# Genotype and transcriptome effects on somatic embryogenesis in *Cryptomeria japonica*

**DOI:** 10.1371/journal.pone.0244634

**Published:** 2020-12-29

**Authors:** Ayako Izuno, Tsuyoshi E. Maruyama, Saneyoshi Ueno, Tokuko Ujino-Ihara, Yoshinari Moriguchi

**Affiliations:** 1 Department of Forest Molecular Genetics and Biotechnology, Forestry and Forest Products Research Institute, Tsukuba, Ibaraki, Japan; 2 Graduate School of Science and Technology, Niigata University, Niigata, Japan; Lovely Professional University, INDIA

## Abstract

Somatic embryogenesis (SE), which is *in vitro* regeneration of plant bodies from somatic cells, represents a useful means of clonal propagation and genetic engineering of forest trees. While protocols to obtain calluses and induce regeneration in somatic embryos have been reported for many tree species, the knowledge of molecular mechanisms of SE development is still insufficient to achieve an efficient supply of somatic embryos required for the industrial application. *Cryptomeria japonica*, a conifer species widely used for plantation forestry in Japan, is one of the tree species waiting for a secure SE protocol; the probability of normal embryo development appears to depend on genotype. To discriminate the embryogenic potential of embryonal masses (EMs) and efficiently obtain normal somatic embryos of *C*. *japonica*, we investigated the effects of genotype and transcriptome on the variation in embryogenic potential. Using an induction experiment with 12 EMs each from six genotypes, we showed that embryogenic potential differs between/within genotypes. Comparisons of gene expression profiles among EMs with different embryogenic potentials revealed that 742 differently expressed genes were mainly associated with pattern forming and metabolism. Thus, we suggest that not only genotype but also gene expression profiles can determine success in SE development. Consistent with previous findings for other conifer species, genes encoding leafy cotyledon, wuschel, germin-like proteins, and glutathione-S-transferases are likely to be involved in SE development in *C*. *japonica* and indeed highly expressed in EMs with high-embryogenic potential; therefore, these proteins represent candidate markers for distinguishing embryogenic potential.

## Introduction

Somatic embryogenesis (SE), which involves *in vitro* development of the bipolar plant body from somatic cells, is an effective means of clonal propagation of forest trees and promote breeding in plantation forestry and the conservation of valuable tree species. The SE-based method has advantages over conventional methods of clonal propagation, such as cutting, grafting, and coppicing, in terms of efficiency and species preservation. Once a proper protocol for culturing is established, SE-based propagation enables indoor production of any number of clonal plants. As juvenile plants derived from somatic embryos can regenerate even after cryopreservation, the long-term preservation of genetic resources and stable supply of saplings is feasible with SE-based propagation. Currently, SE-based propagation is applied on a commercial scale for the forestry of limited number of conifer species in genera such as *Abies*, *Larix*, *Picea*, *Pinus*, and *Pseudotsuga* [1, 2].

The molecular mechanisms underlying SE development remain to be fully elucidated and thus, developing SE protocols has been difficult for many tree species. Based on the gene expression assays and recent genome-wide studies, some gene families that encode SE receptor kinase, leafy cotyledon (LEC), and wuschel (WUS) proteins likely have important functions in SE development [3, 4]. However, it is unclear whether the genetic mechanisms controlling SE development are common across species. Furthermore, the importance of epigenetic regulation, which can also modulate gene reprogramming and determine the embryogenic state of culture cells [5, 6], requires further investigation. Elucidating the molecular mechanisms underlying SE development will help improve current SE protocols, which have often been established through trial and error.

In Japan, *Cryptomeria japonica* D. Don (Cupressaceae) is one of the most important conifer species in the plantation forestry, comprising 4.5 million ha (~45%) of plantation stands [7]. Conventional breeding has depended on cutting of selected trees with superior traits; however, SE-based propagation could improve *C*. *japonica* breeding programs with, for example, genetic engineering. One of the promising applications of genetic engineering in *C*. *japonica* involves establishing male-sterile varieties, which would benefit the ~30% of the Japanese population who are allergic to pollens from *C*. *japonica* plantations [8]. Genetic engineering with SE is theoretically possible in *C*. *japonica* because protocols to induce and maintain somatic embryos have been developed [9–12] and transformation with somatic embryos has been reported [13]. Accumulated genetic data for *C*. *japonica* [e.g., 14–21], as well as a future genome assembly, will also support genetic engineering of varieties with desirable traits.

We recently found a substantial variation in the embryogenesis of calluses among *C*. *japonica* siblings (i.e., different genotypes originated from identical parents) even with the optimum protocols for the *C*. *japonica* SE system [10, 11]. Embryogenic potential cannot be distinguished until somatic embryos actually start developing; such unpredictable variability means that many different genotypes must be maintained and this hinders the industrial application of SE-based propagation for *C*. *japonica*. In the present study, to improve the SE protocols for *C*. *japonica*, we investigated (1) whether the variability in embryogenesis is genetically determined and (2) whether genetic expression profiles differ between embryonal masses with high-embryogenic and low-embryogenic potential. Our results indicate that not only genotypes but also gene expression profiles are associated with the variation in the embryogenic potential. We report some transcriptome markers to discriminate the embryogenic potential in the *C*. *japonica* SE system.

## Materials and methods

### Cell lines

We used six embryogenic *C*. *japonica* cell lines, namely SSD-18, 73, 100, 113, 182, and 352, which were obtained from zygotic embryos excised from immature seeds in July 2016. The seeds were collected from a hybrid offspring produced by ‘Shindai 3’ (maternal) and ‘Suzu 2’ (paternal). The detailed procedures used to obtain embryonal masses (EMs) were described in [22]. The EMs were maintained and proliferated every two weeks with EM medium (S1a Table). Embryogenesis was confirmed at least eight times with the maturation media (S1b Table) before this study began.

### Maturation

In August 2019, 12 EMs (100 mg fresh weight each) per cell line were transferred to Petri dishes (three EMs per dish) with the maturation media (S1b Table). The Petri dishes were maintained in darkness at 25°C. At 5, 7, and 9 weeks after the maturation, the number of normal somatic embryos was recorded and compared between and within cell lines by Tukey’s multiple comparison test.

### RNA isolation

We investigated the gene expression profiles of five EMs from SSD-100, in which we found marked differences in somatic embryos maturation. At 5, 7, and 9 weeks after maturation, 100 mg of tissue was preserved in 200 μl of CTAB buffer at −80°C until RNA extraction. As these EMs originated from an identical cell line (i.e., genotype), sequence polymorphisms in transcripts would not occur among samples. Therefore, the difference in the number of reads mapped on transcript sequences likely reflects differences in expression levels.

Immediately after the frozen tissue solutions had melted at room temperature, the tissues were macerated with a zirconium ball using TissueLyser II (Qiagen). After shaking at 30 Hz for 30 s, the sample tubes were placed on ice to avoid RNA degradation. This procedure was repeated four times. The sample tubes were then incubated at 65°C for 10 min with an additional 200 μl of CTAB buffer (containing 2% CTAB, 40 mM EDTA, 100 mM Tris-HCl (pH 8.0), 2% PVP, 1.4 M NaCl, and 2% 2-mercaptoethanol). Subsequently, the solutions were mixed with a half volume of chloroform/isoamyl alcohol and then centrifuged at 13,000 *g* for 15 min. After collection of approximately 300 μl of the aqueous phase in a new tube, the RNA molecules were precipitated with a quarter volume of 10M LiCl at −20°C for 2 h and subsequently centrifuged at 20,000 *g* and 4°C for 25 min. The pellet was purified using the SV Total RNA Isolation System (Promega) following the manufacturer’s protocol.

### Transcriptome sequencing

The RNA-Seq libraries were prepared using TruSeq Stranded mRNA Library Prep (Illumina) and sequenced with 2 × 100 bp technology in the NovaSeq 6000 System (Illumina). The sequence quality of raw reads was controlled using Trimmomatic (ver. 0.39) [23]. The clean read pairs were aligned on the reference cDNA sequences CJ3006NRE (49,758 transcripts of 39,229 genes expressed in mature leaves, inner bark, and male flowers; [24]) using bowtie2 (ver. 2.3.4.1) [25]. The number of reads was counted for each gene using RSEM (ver. 1.3.2) [26].

We gathered unmapped read pairs, which constituted ~20% (range: 15.7%–26.0%) of input read pairs per sample, and created a new assembly using Trinity (ver. 2.8.4) [27]. Open reading frames (ORFs) in the new transcript assembly were identified using TransDecoder (ver. 5.5.0; https://github.com/TransDecoder/TransDecoder/wiki). Using NCBI BLAST-P (ver. 2.7.1), the homology sequences of the ORFs were searched against 45,644 isoform sequences for plants reposted in SwissProt (downloaded on March 30, 2020) [28]. Using hmmscan in HMMER (ver. 3.2.1; http://hmmer.org/), protein domains in the ORFs were identified by reference to 18,197 Pfam-A domains (ver. 33.0; downloaded on March 30, 2020 [29]). Based on these two annotations, the coding sequences in the new assembly were predicted using TransDecoder (ver. 5.5.0). Short transcript sequences (< 500 bp) were removed. The unmapped reads were aligned on the new transcript assembly using bowtie2 (ver. 2.3.4.1) and the read coverage per gene was counted using RSEM (ver. 1.3.2). The read count data for CJ3006NRE and the new transcript assembly were integrated into a single matrix.

### Differentially expressed genes between EMs with high-embryogenic and low-embryogenic potential

Based on the observed number of embryos, we recognized two out of five EMs (namely, C1 and C5) to have high embryogenic (HE) potential and the remaining (namely, C3, C4, and C6) to have low-embryogenic (LE) potential. In C1 and C5, the number of embryos increased from the 5th to 7th week and decreased from the 7th to 9th week, indicating that the development of new somatic embryos was active at the 5th and 7th weeks but had decelerated by the 9th week. Therefore, we searched genes significantly expressed in HE samples (C1 and C5) at the 5th and 7th weeks relative to expression in the LE samples (C3, C4, and C6). We selected genes with more than one count per million reads in at least two samples. Read count data were normalized based on the trimmed mean of M values normalization method [30]. Differentially expressed genes between HE and LE samples were identified using the *glmQLFit* and *glmQLFTest* functions implemented in the R package edgeR (ver. 3.30.1) [30]. Genes with less than 0.05 false discovery rate and more than 2 log-fold-change were considered significant.

Gene functions enriched in the target genes were identified using the R package topGO with the “elim” algorithm (ver. 2.40.0) [31]. Gene Ontology (GO) terms with *p* < 0.01 were considered to be significant. For enrichment analysis, we annotated the reference cDNA sequences CJ3006NRE with the SwissProt plant isoform sequence data (the same data used to annotate the new assembly of unmapped reads; see above) and then obtained GO terms (S3 Table).

### Validation of differentially expressed genes by quantitative PCR

Among the differentially expressed genes identified by RNA-Seq, the expression levels of six genes (S5 Table) were validated by quantitative PCR (qPCR). Alpha-tubulin (AT1G50010 in the *Arabidopsis thaliana* genome) was selected for a reference housekeeping gene, because it is suitable for qPCR assays during somatic embryo development in conifer species [32]. Based on the reciprocal best hits between CJ3006NRE and TAIR10, a *C*. *japonica* transcript CJt088185 was assumed to be the most homologous with AT1G50010 and used for the subsequent primer design.

We first identified exon-exon junctions on template transcript sequences based on the alignment with the homologous gene sequences in TAIR10 (S5 Table). Primer pairs over the exon-exon junctions were designed using Primer-BLAST [33], with 80–150 bp of product size, 57–63°C (optimal: 60°C) of annealing temperature, 17–25 bp (optimal: 20 bp) of primer length, and 45–60°C of primer GC content. Primer specificity was checked through *in silico* PCR with a total of 81,353 *C*. *japonica* transcript sequences used in this study (S3 and S4 Tables).

We performed qPCR for a total of 10 EM samples, which are composed of five EMs of SSD-100 collected at the 5th and 7th week after maturation and used for RNA-Seq. After removing genomic DNA from ca. 500 ng of total RNA using gDNA Remover, cDNA was synthesized using ReverTra Ace qPCR RT Master Mix (TOYOBO). Each of six target and one reference loci was amplified from the cDNA template in triplicate of 10 μl reaction solution including 5–10% of cDNA, 1×KOD SYBR qPCR Mix (TOYOBO), and 0.2 μM each of forward and reverse primers. Using LightCycler 480 (Roche), the intensity of SYBR Green I was measured during PCR, which was composed of an initial step at 98°C for 2 min and 40 cycles of denaturation at 98°C for 10 sec, annealing at 60°C for 10 sec, and extension at 68°C for 30 sec. After the amplification step, a denaturation at 95°C for 5 sec and an annealing at 65°C for 1 min followed, then a melting curve analysis was carried out from 65°C to 95°C with continuous fluorescent measurements at 5°C interval. The absence of primer dimers was confirmed by the reactions without cDNA (i.e., negative controls). The mean of cycle thresholds (Ct) was identified using 2nd derivative maximum method for each reaction, then relative expression level among the 10 samples was calculated for each locus with delta-delta Ct method, in which the expression level was normalized with that in C4 from the 7th week. The difference in gene expression level between EMs was examined using Tukey’s multiple comparison test.

## Results

### SE development

**Fig 1:**
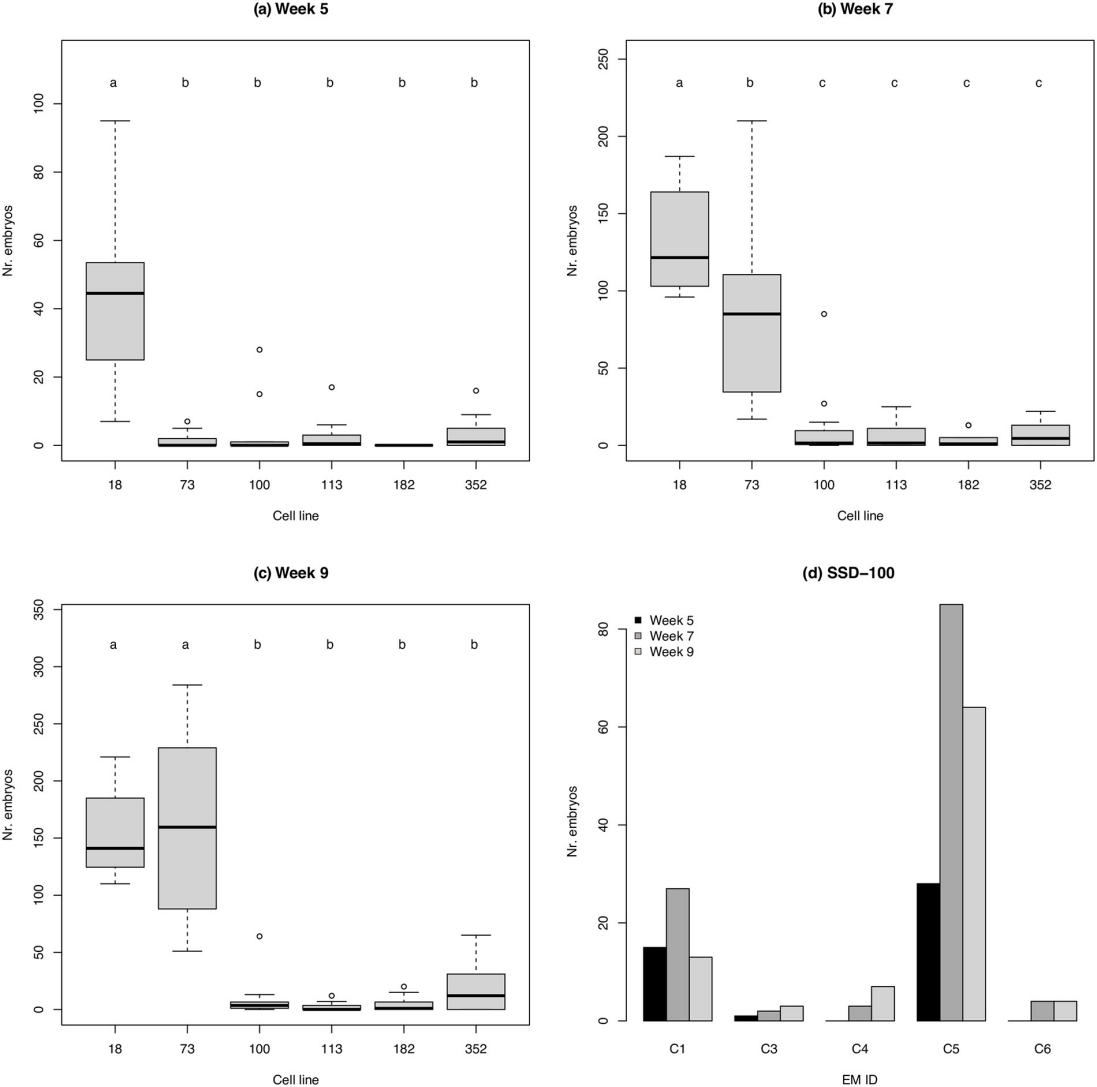
**(a–c)** The number of embryos observed in six *Cryptomeria japonica* cell lines at (a) 5, (b) 7, and (c) 9 weeks after maturation. Twelve embryonal masses (EMs) were cultured per cell line. Means with different letters are significantly different (Tukey’s multiple comparison test, *p* < 0.01). **(d)** The number of embryos observed in five EMs of the cell line SSD-100 at 5, 7, and 9 weeks after maturation. These EMs were used for RNA-Seq.

The number of embryos developed from the 5th to 9th week varied between cell lines. SSD-18 and 73 showed higher capacities for developing somatic embryos than the other four cell lines during 5–9 and 7–9 weeks after maturation, respectively (*p* < 0.01, Fig 1a–1c). In some cell lines, namely SSD-100 and 113, the mean number of embryos developed during the experiment period was significantly different among EMs within cell lines (*p* < 0.01, S1 Fig).

**Fig 2:**
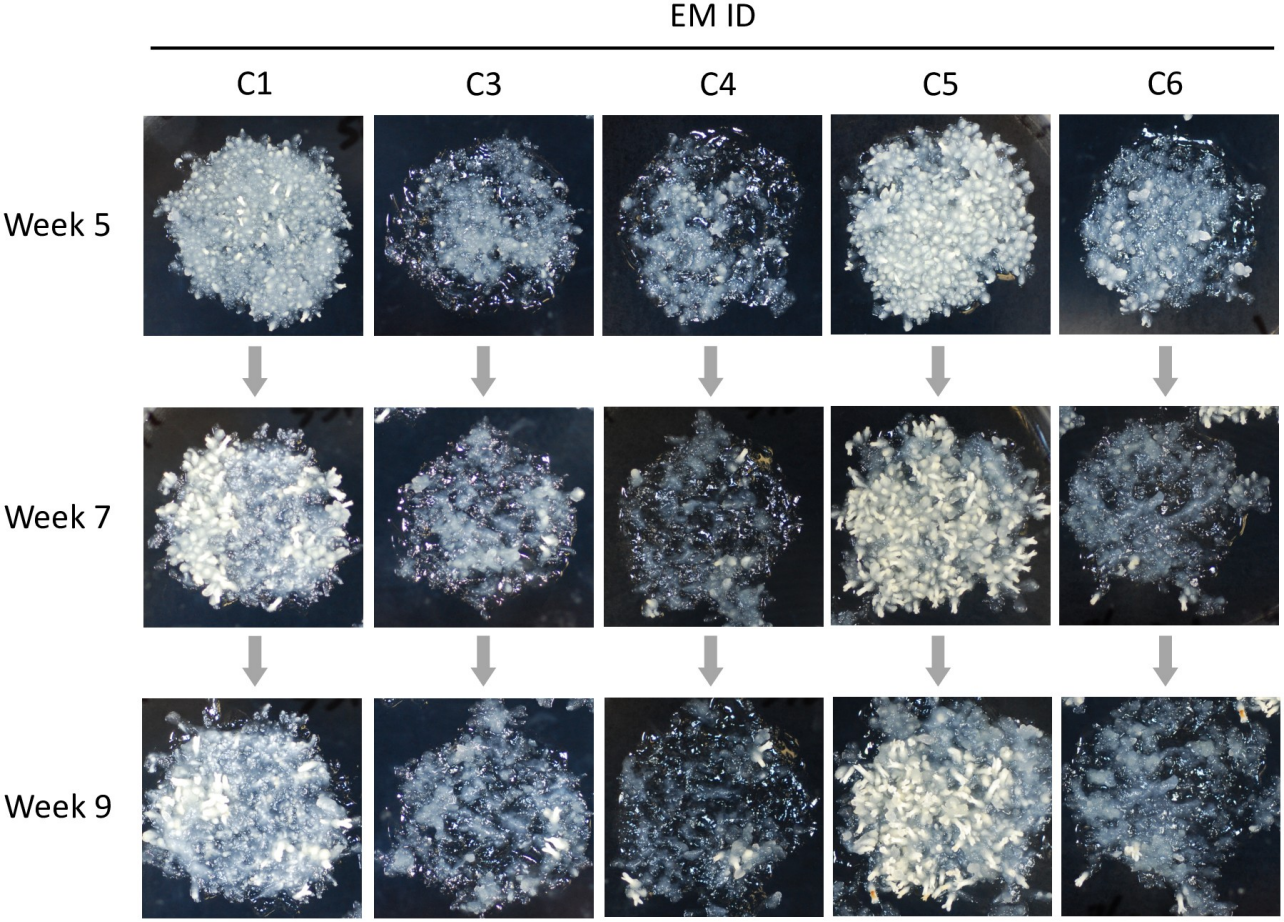
The five *Cryptomeria japonica* embryonal masses (EMs) induced from an identical cell line SSD-100 at 5, 7, and 9 weeks after maturation. The capacity to develop somatic embryos are different between EMs.

We found the five EMs from SSD-100 to be suitable to assay gene expressions because they showed a marked difference in the capacity to develop somatic embryos in spite of their shared genotype (Fig 2). C1 and C5 developed more than10 embryos throughout the experimental period, whereas C3, C4, and C6 did not (Fig 1d). Because we collected some normal somatic embryos at each time point, the number of embryos could subsequently decrease unless embryogenesis was actively maintained. In C1 and C5, new somatic embryos appeared at the 5th and 7th week, but did not appear at the 9th week. Based on these observations, we considered C1 and C5 at the 5th and 7th week to have high embryogenic potential.

### Transcriptome sequencing

We obtained 20–37 × 10^6^ read pairs (4.1–7.5 × 10^9^ bases) per sample (S2 Table). Raw data are deposited on DDBJ DRA (Accession number: DRA010541). After filtering, 3.98–7.33 × 10^9^ bases remained per sample and 74–84% of these were properly mapped onto CJ3006NRE. *De novo* assembly of unmapped reads generated 204,943 transcript sequences. Based on a homology search against SwissProt and Pfam-A, 78,415 isoform sequences of 25,364 genes were predicted. After removing short (< 500 bp) sequences, 31,595 isoform sequences of 12,073 genes were used for subsequent analyses. This assembly is deposited in DDBJ (Accession number: ICQR01000001–ICQR01031595) and the annotation is shown in S4 Table.

### Differentially expressed genes between EMs with high-embryogenic and low-embryogenic potential

At the 5th and 7th week, 742 genes were significantly upregulated in HE samples compared with LE samples. The 742 genes upregulated in HE samples included some candidate genes encoding such as leafy cotyledon (LEC; *g28713*; S3 Table), wuschel (WUS; *g27218* and *g34977*; S3 Table), and germin-like protein (GLP; g*31725* and *g38183*; S3 Table), as well as glutathione-S-transferase (GST; seven genes including *g3323* and *TRINITY_DN4815_c0_g1*; S3 and S4 Tables). Consistent with the developmental stages in our experiment, we found ten genes encoding late embryogenesis abundant proteins. GO enrichment analysis showed that the 742 genes were significantly involved in the development of the plant body, especially specifications of polarity, and metabolic processes including the biosynthesis of flavonoids, gibberellins, and brassinosteroids (Table 1).

**Table 1:** Over-represented Gene Ontology (GO) terms in the 742 genes upregulated in high embryogenic *Cryptomeria japonica* embryonal masses.

Category	GO ID	Term	*p*
Development			
	GO:0010158	Abaxial cell fate specification	2.E-05
	GO:0080060	Integument development	3.E-04
	GO:1902183	Regulation of shoot apical meristem development	8.E-04
	GO:0009943	Adaxial/abaxial axis specification	3.E-03
	GO:0010073	Meristem maintenance	4.E-03
	GO:0009944	Polarity specification of adaxial/abaxial axis	4.E-03
	GO:0099402	Plant organ development	7.E-03
	GO:1901342	Regulation of vasculature development	8.E-03
Metabolic process			
	GO:0009813	Flavonoid biosynthetic process	3.E-09
	GO:0009686	Gibberellin biosynthetic process	3.E-07
	GO:0016131	Brassinosteroid metabolic process	5.E-07
	GO:0016042	Lipid catabolic process	8.E-05
	GO:0006110	Regulation of glycolytic process	2.E-04
	GO:1901959	Positive regulation of cutin biosynthetic process	3.E-04
	GO:0009699	Phenylpropanoid biosynthetic process	7.E-04
	GO:0010345	Suberin biosynthetic process	8.E-04
	GO:0033473	Indoleacetic acid conjugate metabolic process	9.E-04
	GO:0042744	Hydrogen peroxide catabolic process	1.E-03
	GO:0006694	Steroid biosynthetic process	1.E-03
	GO:0016132	Brassinosteroid biosynthetic process	2.E-03
	GO:2000762	Regulation of phenylpropanoid metabolic process	2.E-03
	GO:0046463	Acylglycerol biosynthetic process	3.E-03
	GO:0042761	Very long-chain fatty acid biosynthetic process	5.E-03
	GO:0009690	Cytokinin metabolic process	7.E-03
	GO:0046189	Phenol-containing compound biosynthetic …	7.E-03
	GO:0010430	Fatty acid omega-oxidation	7.E-03
	GO:0006571	Tyrosine biosynthetic process	8.E-03
	GO:0009696	Salicylic acid metabolic process	8.E-03
	GO:1900378	Positive regulation of secondary metabolite biosynthetic process	8.E-03
Response to stimulus			
	GO:0009744	Response to sucrose	5.E-04
	GO:0043481	Anthocyanin accumulation in tissues in response to UV light	6.E-04
	GO:0009725	Response to hormone	2.E-03
	GO:1901700	Response to oxygen-containing compound	3.E-03
	GO:0033993	Response to lipid	3.E-03
	GO:0009958	Positive gravitropism	9.E-03
Transport			
	GO:0080170	Hydrogen peroxide transmembrane transport	6.E-06
	GO:0015840	Urea transport	1.E-05
	GO:0071577	Zinc ion transmembrane transport	3.E-04
	GO:0008643	Carbohydrate transport	5.E-03
	GO:0006833	Water transport	9.E-03
Others			
	GO:0010268	Brassinosteroid homeostasis	5.E-08
	GO:0070207	Protein homotrimerization	2.E-06
	GO:0010431	Seed maturation	3.E-05
	GO:0036290	Protein trans-autophosphorylation	3.E-04

### Validation of differentially expressed genes by quantitative PCR

**Fig 3:**
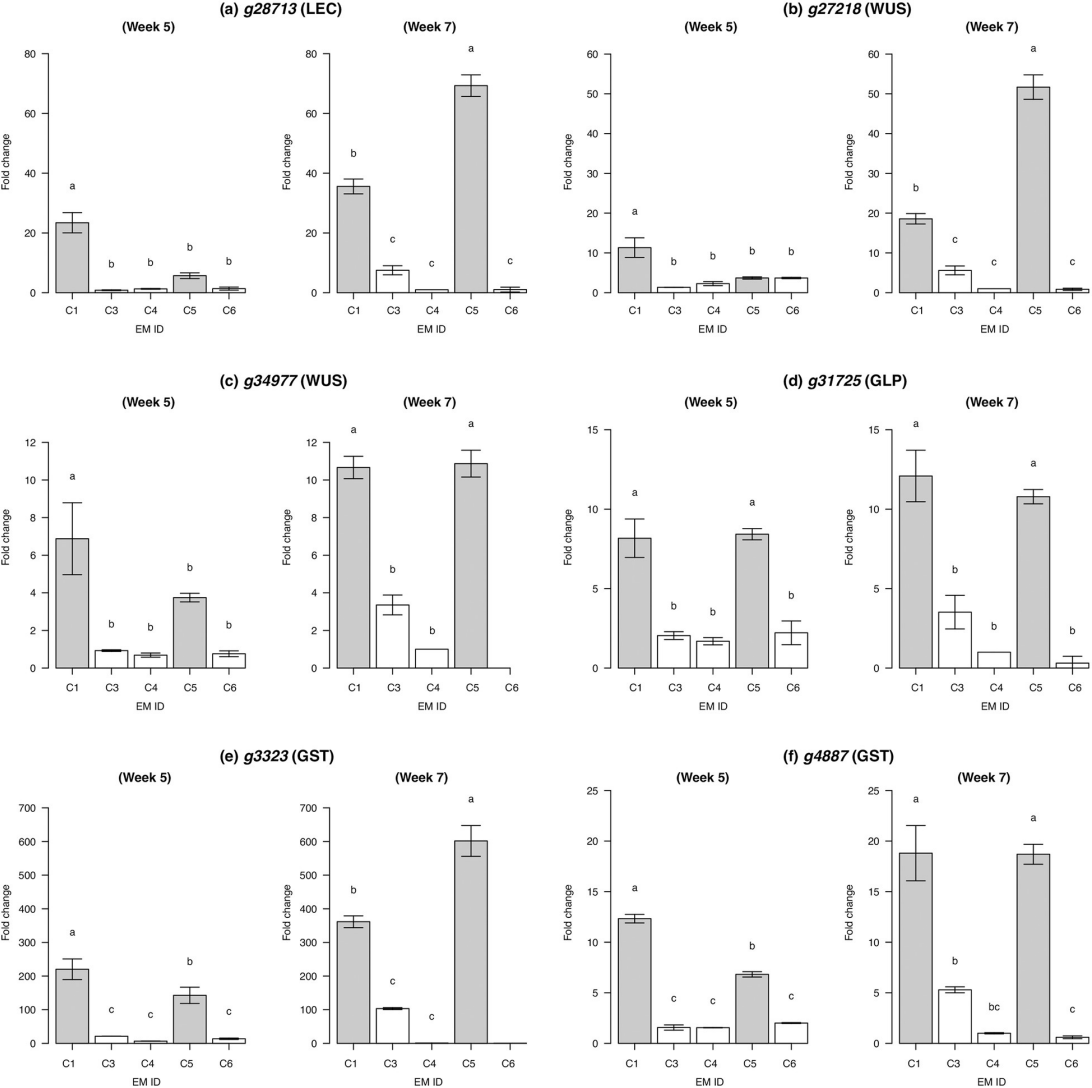
Relative expressions of putatively embryogenesis-related genes in *Cryptomeria japonica*. Expressions of six genes encoding (a) leafy cotyledon (LEC), (b, c) wuschel (WUS), (d) germin-like protein (GLP), and (e, f) glutathione-S-transferase (GST) were quantitated. For each gene, expression levels were normalized with that in C4 from the 7th week. Means with different letters are significantly different (Tukey’s multiple comparison test, *p* < 0.01). Samples with high embryogenic potential (C1 and C5) were indicated in gray. In C6 from the 7th week, the expressions of (c) *g34977* and (e) *g3323* were not quantified due to the low expressions.

qPCR assays showed that HE samples (C1 and C5) more highly expressed six candidate genes compared to LE samples (C3, C4, and C6) except for the C5 at the 5th week, where the expression levels of three genes, namely *g28713* (LEC), *g27218* (WUS), and *g34977* (WUS), were not significantly different from LE samples (Fig 3). Clear difference in expression levels between HE and LE samples was particularly found at the 7th week, consistent with the increased number of somatic embryos (Figs 1d and 2).

## Discussion

Our results showed that the embryogenesis of *C*. *japonica* largely differs among cell lines (i.e., genotypes). Although we examined six cell lines with embryogenic potential, only two (SSD-18 and 73) showed stable embryogenesis (Fig 1a–1c). This is consistent with the varying potential of cell lines for forming embryos reported in other conifer species such as *Pinus radiata* [34]. Besides the variation among cell lines, we showed that embryogenesis differs within cell lines (S1 Fig). Given that all the EMs within cell lines share the same genomic background, embryogenic potential may also depend on gene expression regulation of relevant genes. Intrinsic factors, such as the totipotency of cells transplanted to maturation media, and extrinsic factors, including the concentration of the medium available for EMs, may affect the signaling pathways of gene expressions. The variation we observed in embryogenesis between and within cell lines confirms that a substantial number of cell lines as well as replicated EMs should be maintained to ensure a stable supply of *C*. *japonica* somatic embryos. These findings critically indicate the necessity of a means to identify embryogenic potential at certain developmental stages.

We found that gene expression profiles clearly differed between the HE and LE samples at the 5th and 7th weeks after maturation, during which somatic embryos developed in abundance. GO enrichment analysis suggested that genes upregulated in HE samples were involved in the pattern forming and metabolism required to develop somatic embryos (Table 1). In the 742 upregulated genes, we found some candidate genes: leafy cotyledon (LEC), wuschel (WUS), germin-like protein (GLP), and glutathione-S-transferase (GST) genes. LEC genes are required for the specification of cotyledon identity and the completion of embryo maturation [35]. The WUS gene family acts to maintain the stem cell population in shoot apical meristems [36, 37], root apical meristems [38], and cambial meristems [39, 40]. Both LEC and WUS proteins play central roles in the auxin signaling pathway and regulate somatic cells for embryogenic development [41]. In *Arabidopsis thaliana*, LEC genes are activated by WUS genes to promote SE [42]. The high expression of LEC and WUS genes during SE development has also been confirmed in other conifer species (*Picea* and *Pinus* species; [34, 43, 44]). The GLP gene family is thought to mediate the initiation of embryo germination in wheat [45], *Arabidopsis* [46], and cotton [47]. In *Pinus* and *Picea*, GLPs likely function during the early stages of SE induction, as suggested by proteomic and transcriptomic studies [48–50]. GST enzymes are known to act under stress conditions and to minimize oxidative damage on cells. The transcripts have been detected in somatic embryos in *Chicorium* [51] and wheat [52].

According to a review by Mahdavi-Darvari et al. [3], LEC, WUS, GLP, and GST genes are good candidate markers for embryogenic potential. As our qPCR results validated the different expressions between HE and LE samples (Fig 3), the primers (S5 Table) can be used as appropriate markers to discriminate embryogenic potential in *C*. *japonica*. Identifying the expression profiles of these genes in the early stages of SE development will further inform the effectiveness of these genes as markers. Besides the focus on gene expression, studies on epigenetic [53] and proteome processes [54] could provide additional insights to improve the efficient collection of quality somatic embryos.

## Supporting information

S1 Table Chemical composition of (a) maintenance-proliferation medium and (b) maturation medium. (DOCX)

S2 Table The number of RNA-Seq reads processed. (DOCX)

S3 Table Gene annotations for CJ3006NRE. (XLSX)

S4 Table Gene annotations for the transcript sequence assembly of reads not aligned on the CJ3006NRE. (XLSX)

S5 Table Primer information for quantitative PCR. (XLSX)

S1 Fig The number of somatic embryos obtained in each embryonal mass at the 5th, 7th, and 9th week after maturation. Means with different letters are significantly different (Tukey’s multiple comparison test, *p* < 0.01).
(TIF)
