# Supplementary material for: Genotype and transcriptome effects on somatic embryogenesis in Cryptomeria japonica
Source: PLoS One. 2020 Dec 29;15(12):e0244634. doi: 10.1371/journal.pone.0244634 (PMC7771663; doi:10.1371/journal.pone.0244634)
Supplement: S1 Table — (DOCX) [file pone.0244634.s001.docx]

**S1 Table** Chemical composition of **(**a) maintenance-proliferation medium and (b) maturation medium.

(a)

| Compound | Amounts |  |
| --- | --- | --- |
| KNO_3_ | 500 | [mg/L] |
| MgSO_4_・7H_2_O | 250 |  |
| CaCl_2_・2H_2_O | 37.5 |  |
| Ca(NO_3_)_2_・4H_2_O | 30 |  |
| NaNO_3_ | 30 |  |
| KH_2_PO_4_ | 35 |  |
| NaH_2_PO4・2H_2_O | 80 |  |
| KCl | 40 |  |
| MnSO_4_・4H_2_O | 10 |  |
| H_3_BO_3_ | 20 |  |
| ZnSO_4_・7H_2_O | 12.5 |  |
| KI | 0.5 |  |
| CuSO_4_・5H_2_O | 1.2 |  |
| Na_2_MoO_4_・2H_2_O | 0.1 |  |
| CoCl_2_・6H_2_O | 0.1 |  |
| FeSO_4_・7H_2_O | 15 |  |
| NaEDTA | 20 |  |
| Thiamine HCl | 2.5 |  |
| Pyridoxine HCl | 0.25 |  |
| Nicotinic acid | 2.5 |  |
| Glycine | 2.5 |  |
| myo-Inositol | 500 |  |
| Casein Acid Hydrolysate | 0 |  |
| L-glutamine | 1500 |  |
| Sucrose | 30000 |  |
| Gelrite | 3000 |  |
| 2,4-D | 3 | [μM] |
| BAP | 1 |  |
| pH 5.8 |  |  |

(b)

| Compound | Amounts |  |
| --- | --- | --- |
| KNO_3_ | 1000 | [mg/L] |
| MgSO_4_・7H_2_O | 500 |  |
| CaCl_2_・2H_2_O | 75 |  |
| Ca(NO_3_)_2_・4H_2_O | 60 |  |
| NaNO_3_ | 60 |  |
| KH_2_PO_4_ | 70 |  |
| NaH_2_PO4・2H_2_O | 160 |  |
| KCl | 80 |  |
| MnSO_4_・4H_2_O | 20 |  |
| H_3_BO_3_ | 40 |  |
| ZnSO_4_・7H_2_O | 25 |  |
| KI | 1 |  |
| CuSO_4_・5H_2_O | 2.4 |  |
| Na_2_MoO_4_・2H_2_O | 0.2 |  |
| CoCl_2_・6H_2_O | 0.2 |  |
| FeSO_4_・7H_2_O | 30 |  |
| NaEDTA | 40 |  |
| Thiamine HCl | 5 |  |
| Pyridoxine HCl | 0.5 |  |
| Nicotinic acid | 5 |  |
| Glycine | 5 |  |
| myo-Inositol | 1000 |  |
| L-glutamine | 2000 |  |
| L-asparagine | 1000 |  |
| L-arginine | 500 |  |
| L-citrulline | 79 |  |
| L-ornithine | 76 |  |
| L-lysine | 55 |  |
| L-alanine | 40 |  |
| L-proline | 35 |  |
| Maltose | 30000 |  |
| Polyethylene glycol 6000 | 175000 |  |
| Activated charcoal | 2000 |  |
| Gelrite | 3300 |  |
| Abscisic acid | 100 | [μM] |
| pH 5.8 |  |  |
