# Supplementary material for: Genotype and transcriptome effects on somatic embryogenesis in Cryptomeria japonica
Source: PLoS One. 2020 Dec 29;15(12):e0244634. doi: 10.1371/journal.pone.0244634 (PMC7771663; doi:10.1371/journal.pone.0244634)
Supplement: S2 Table — (DOCX) [file pone.0244634.s002.docx]

**S2 Table** Number of RNA-Seq reads processed.

| EM ID | Week | Nr. read pairs obtained | Nr. read pairs after QC | Nr. read pairs mapped to CJ3006NRE | (%) |
| --- | --- | --- | --- | --- | --- |
| C1 | 5 | 24,834,061 | 24,461,180 | 19,362,016 | 79.15 |
| C1 | 7 | 35,985,685 | 35,306,214 | 26,785,967 | 75.87 |
| C1 | 9 | 32,317,730 | 31,757,592 | 24,423,537 | 76.91 |
| C3 | 5 | 33,881,438 | 33,246,509 | 28,011,292 | 84.25 |
| C3 | 7 | 35,761,936 | 35,100,253 | 28,650,155 | 81.62 |
| C3 | 9 | 33,318,511 | 32,720,600 | 25,986,982 | 79.42 |
| C4 | 5 | 28,170,708 | 27,630,363 | 23,206,462 | 83.99 |
| C4 | 7 | 20,134,832 | 19,779,555 | 16,470,792 | 83.27 |
| C4 | 9 | 25,705,753 | 25,255,983 | 18,918,239 | 74.91 |
| C5 | 5 | 30,175,427 | 29,677,947 | 24,353,841 | 82.06 |
| C5 | 7 | 28,118,978 | 27,669,424 | 20,473,348 | 73.99 |
| C5 | 9 | 27,744,513 | 27,265,144 | 20,304,192 | 74.47 |
| C6 | 5 | 37,038,969 | 36,386,944 | 30,627,236 | 84.17 |
| C6 | 7 | 23,921,988 | 23,575,819 | 19,835,895 | 84.14 |
| C6 | 9 | 31,317,407 | 30,757,820 | 24,119,848 | 78.42 |
