# Supplementary figures and images for: Genotype and transcriptome effects on somatic embryogenesis in Cryptomeria japonica
Source: PLoS One. 2020 Dec 29;15(12):e0244634. doi: 10.1371/journal.pone.0244634 (PMC7771663; doi:10.1371/journal.pone.0244634)

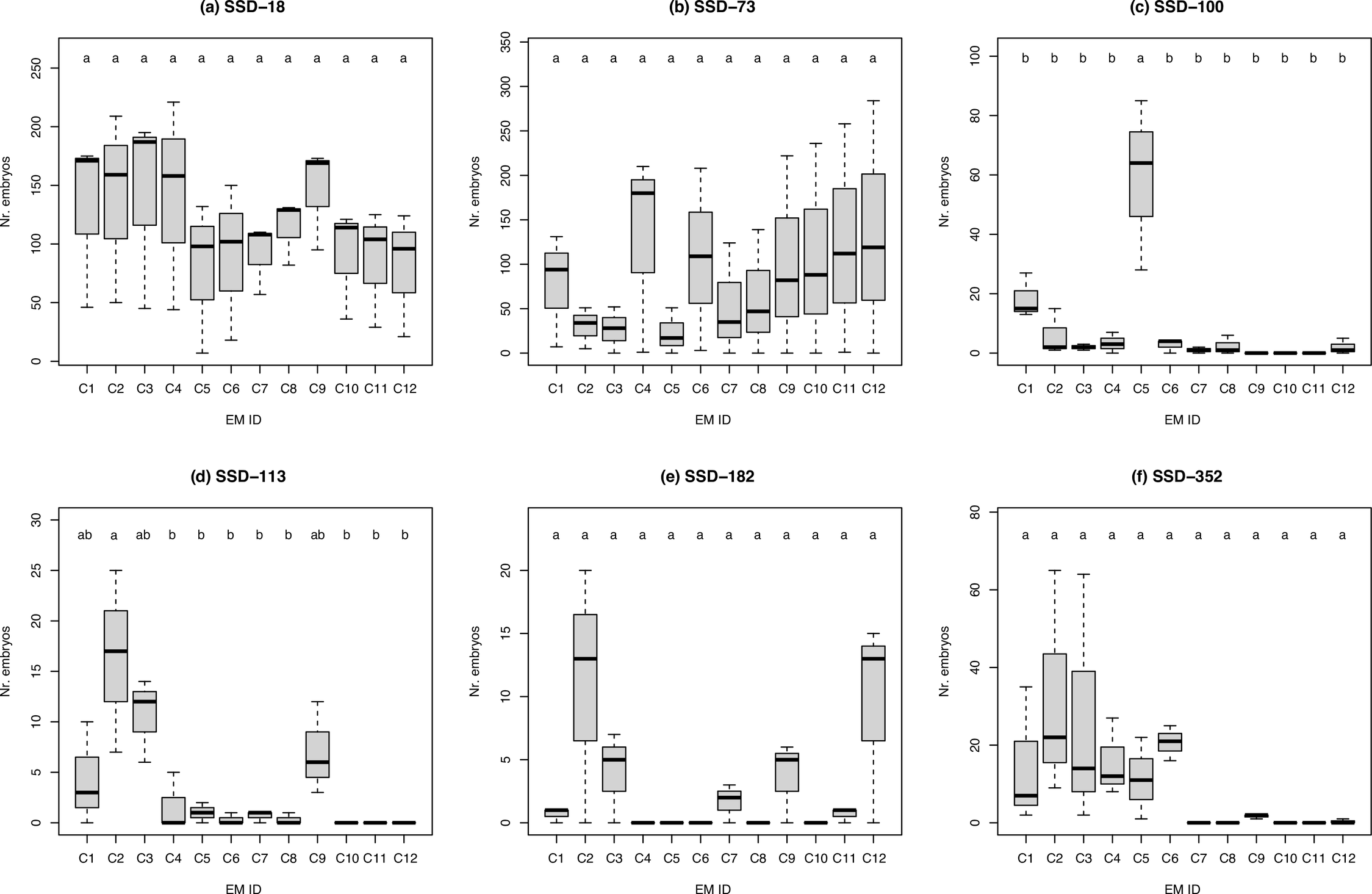

Supplement: S1 Fig — Means with different letters are significantly different (Tukey’s multiple comparison test, p < 0.01). (TIF) [file pone.0244634.s006.tif]
